# ATTNFNET: feature aware depth-to-pressure translation with cGAN training

**DOI:** 10.3389/fmedt.2025.1621922

**Published:** 2025-09-16

**Authors:** Neevkumar Manavar, Hanno Gerd Meyer, Joachim Waßmuth, Barbara Hammer, Axel Schneider

**Affiliations:** ^1^ Faculty of Engineering and Mathematics, Bielefeld University of Applied Sciences, Bielefeld, Germany; ^2^ Faculty of Technology, CITEC, Bielefeld University, Bielefeld, Germany

**Keywords:** patient monitoring, generative network, contact pressure prediction, image translation, deep neural network, transformer

## Abstract

Excessive pressure and shear forces on bedridden patients can lead to pressure injuries, particularly on those with existing ulcers. Monitoring pressure distribution is crucial for preventing such injuries by identifying high-risk areas. To address this challenge, we propose Attention Feature Network (AttnFnet), a self-attention-based deep neural network that generates pressure distribution maps from single-depth images using Conditional Generative Adversarial Network (cGAN) training. We introduce a mixed-domain SSIML2 loss function, combining structural similarity and pixel-level accuracy, along with adversarial loss, to enhance the prediction of pressure distributions for subjects lying in a bed. Evaluation results from the benchmark dataset demonstrate that the AttnFnet outperforms existing methods in terms of Structural Similarity Index Measure (SSIM) and quality analysis, providing accurate pressure distribution estimation from a single depth image.

## 1 Introduction

Image processing techniques have become integral to advancements in medical diagnostics and patient care. Transformer-based model architectures, such as those foundational in Natural Language Processing (NLP) (1) have been successfully adapted for image classification and segmentation tasks (2, 3). However, these models typically require large datasets and significant computational resources to learn global attention patterns and image encodings. This limitation poses challenges in medical applications, where data availability and computational efficiency are critical.

Alternatively, Fully Convolutional Network (FCN)-based models offer computationally less intensive solutions and can provide superior feature representations in the context of limited resources and datasets (4). Despite these advancements, learning image representations using Convolutional Neural Network (CNN) remains complex when attempting to capture global context effectively. Incorporating attention mechanisms with CNN can address this challenge by focusing on relevant features across the entire image (5). Inspired by the original transformer architecture (1) and conditional adversarial training (6), we propose the Attention Feature Network (AttnFnet), a novel model that leverages a convolutional architecture to project image features while employing transformer-like attention mechanisms to obtain global feature context. Our model processes images through 12 transformer layers to generate encodings in a latent space, followed by deconvolution with skip connections back to the image space.

A specific use case in medical applications is studied using AttnFnet. Pressure ulcers pose a significant risk to bedridden patients, often leading to severe complications if not addressed promptly (7). Conventional monitoring methods can be resource-intensive or lack real-time capabilities. By predicting pressure distributions from depth images captured by an overhead camera, our approach offers a non-invasive, efficient tool for continuous patient monitoring. Our experimental results demonstrate that AttnFnet effectively predicts pressure distributions, potentially aiding in timely interventions to reposition patients and prevent pressure injuries.

The motivation for AttnFnet is to capture contextual features in depth images, particularly around pressure-sensitive areas at risk of developing pressure ulcers. This architecture is designed to balance computational efficiency and predictive performance, addressing the limitations of large-scale transformer-based sequence-to-sequence models, which are resource-intensive, and Fully Convolutional Network (FCN)-based encoder-decoder architectures, which often struggle with capturing long-range dependencies. The proposed Structural Similarity Index Measure L2 norm (SSIML2) loss function enables the model to minimize Mean Squared Error (MSE) more effectively than standard L2 loss alone. Additionally, the inclusion of cGAN loss constrains the network to generate contextually relevant outputs, enhancing the fidelity of the predicted pressure maps rather than promoting image diversity.

We evaluated our model’s performance on depth-to-pressure image translation tasks using a publicly available benchmark dataset (8), with the U-Net architecture (9), and previous state-of-the-art BPBnet, and BPWnet (10) as baselines for comparison. Our results indicate that AttnFnet demonstrates promising performance in this specific medical application and shows potential for broader image translation tasks.

This study focuses on critical medical applications, trained on publicly available supine and lateral depth-pressure data, and possibly pinpoint high-risk tissue-loading zones in real time, thereby enabling early off-loading interventions in long-term-care and home settings.

## 2 Related work

### 2.1 Image generation

Since the introduction of Generative Adversarial Network (GAN)s by Goodfellow et al. (11), image generation has gained significant attention in the research community. FCN have emerged as foundational architectures for many GAN-based generation tasks due to their ability to effectively capture spatial hierarchies. Over the years, numerous GAN variants have been proposed for image generation, each enhancing different aspects of the model’s capabilities. Noteable examples include CycleGAN (12), StarGAN (13), Least Squares GAN (14), StyleGAN (15), DCGAN (16), and cGAN (17).

These advancements have paved the way for more sophisticated image translation tasks. For instance, Isola et al. (6) demonstrated the effectiveness of conditional GANs for image-to-image translation tasks. Our proposed model builds upon these foundations by leveraging transformer-based conditional GAN training with a mixed-domain loss function to translate depth images into pressure distribution maps.

### 2.2 CNN architecture

CNNs are foundational models for vision tasks, first introduced by Lecun et al. (18). Their ability to learn hierarchical visual features established them as state-of-the-art for a wide range of vision applications. Prominent models such as ImageNet (19), VGGNet (20), ResNet (21), and MobileNet (22) have employed FCN architectures to capture fine-grained image features, becoming foundational in tasks like image recognition and object detection. In the domain of semantic segmentation, the work by Ronneberger et al. (9) made a significant contribution to FCN-based architectures. The success of U-Net in semantic segmentation and image translation has rapidly established it as a state-of-the-art model.

Our proposed model builds upon a CNN-based architecture and utilizes CNNs to upscale latent representations to pixel space. It leverages the computational efficiency of CNNs in vision tasks to provide an effective and efficient mechanism for upscaling latent features. This study compares the performance of the proposed method with the FCN based U-Net model.

### 2.3 Vision transformer

The introduction of transformers by Vaswani et al. (1) marked a paradigm shift in Natural Language Processing (NLP). The success of transformers in sequence-to-sequence tasks inspired their adaptation to computer vision, leading to the development of Vision Transformer (ViTs) (2). ViTs utilize self-attention mechanisms to capture long-range dependencies in images, proving particularly effective in global feature extraction (23). Subsequent works have explored transformer architectures for various image processing tasks, including image generation and segmentation (24–26).

Kirillov et al. (3) and Zheng et al. (27) extended the transformer capabilities by combining a transformer capabilities with CNNs for segmentation tasks. The proposed model leverages a hybrid transformer-CNN architecture, utilizing CNN layers both in patch projection and as part of the feed-forward network. Additionally, it incorporates skip connections between the encoder and decoder, enhancing information flow and feature retention across the network.

As shown by Raghu et al. (23), ViTs maintain robust feature representations through attention, and transfer learning can significantly accelerate training. In line with these findings, our model employs pre-trained weights from the Segment Anything Model (SAM) (3) to initialize training and hence facilitating efficient convergence and improved performance.

### 2.4 Inferring pressure distribution

Several studies have focused on predicting pressure injuries in hospitalized patients. These studies have utilized statistical models and machine learning techniques to identify risk factors such as body mass index, age, gender, and comorbidities that influence the likelihood of developing pressure injuries (28–31). While effective in risk stratification, these approaches do not provide spatially resolved information on when or where a pressure injury might occur. Hence, understanding body pressure distribution offers deeper insights into the specific locations at risk of pressure ulcer development. Clever et al. (10) utilized BPBnet and BPWnet to predict body pressure distribution using a depth camera, demonstrating the potential of non-invasive monitoring techniques.

Building upon this concept, our approach leverages a transformer-based GAN architecture trained on real-world data with various human poses (8) to predict pressure distributions from depth images. Unlike prior methods, our model incorporates attention mechanisms to improve results on pressure-sensitive areas and adversarial training to enhance prediction accuracy and spatial distribution.

## 3 Methods

This section provides a detailed description of the proposed AttnFnet architecture, training objectives, training strategy, and evaluation metrics. We begin by outlining the structure of the AttnFnet model, including its image encoder, bottleneck layer, and decoder, and then explain how the model is trained using a conditional GAN framework. We also describe the metrics used to evaluate its performance in terms of both pixel-level accuracy and perceptual quality.

### 3.1 AttnFnet architecture

**Figure 1:**
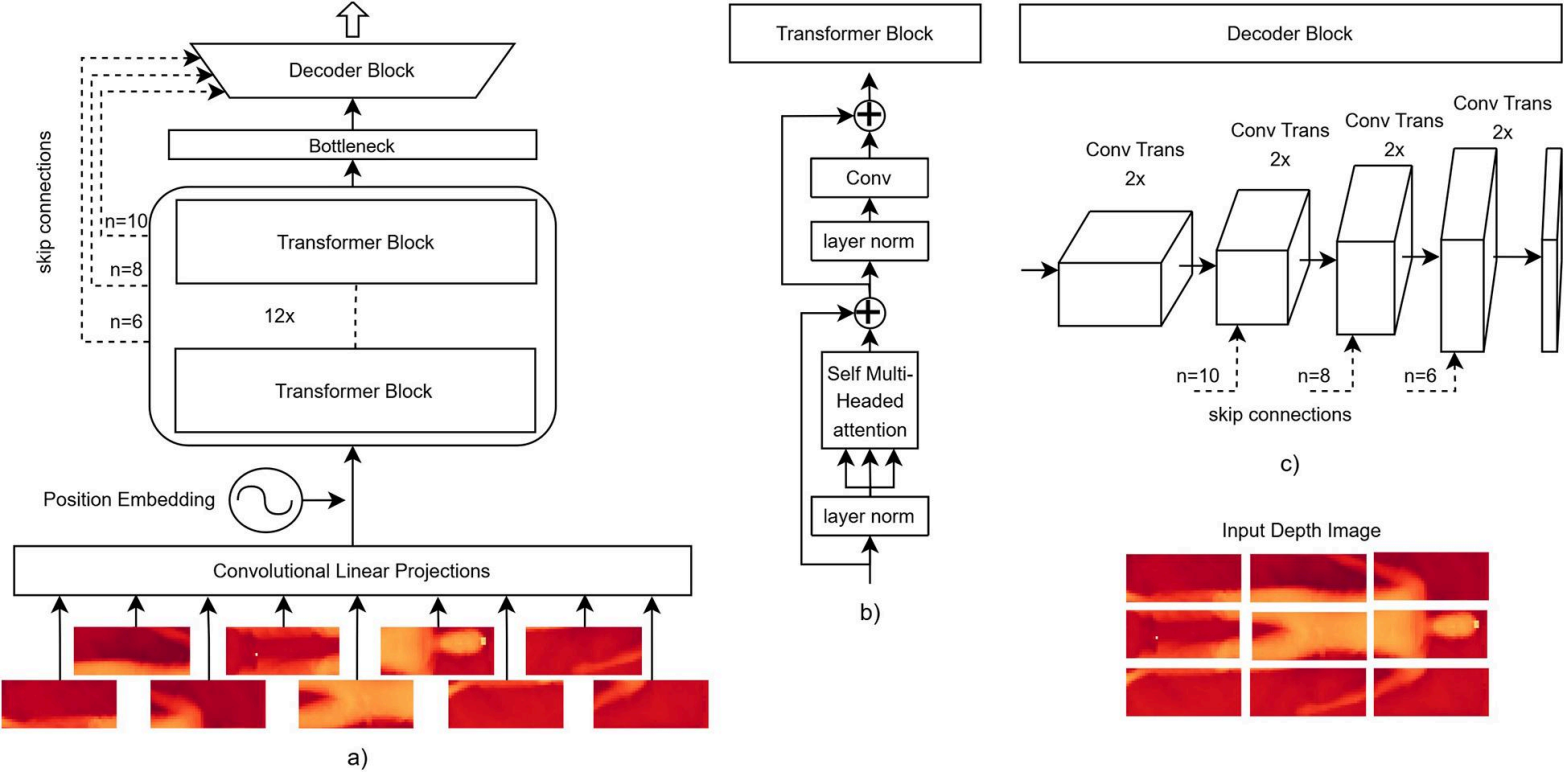
Schematic representation of the AttnFnet model architecture. **(A)** An example architecture for a 128×54 input image. The input image is projected to a 712-dimensional embedding via a convolution operation. Positional embeddings are added to these projections before being processed by the transformer block, and the output is passed through the decoder block with skip connections. **(B)** Transformer block, where the input undergoes a standard multi-head self-attention mechanism followed by convolutional projections. **(C)** Decoder block schematic, where the output from the transformer encoder passes through multiple up-convolution layers, progressively increasing resolution until the desired output size is reached, with skip connections added to the deconvolution blocks. n=6, n=8, and n=10 indicate the number of transformer blocks whose output is used.

The AttnFnet architecture is designed to translate depth into pressure distribution maps. Figure 1 describes overall architecture and it consist of three primary components: 1. an *image encoder* that encodes the image into a latent space, 2. a *bottleneck layer* that reduces computational complexity while preserving crucial features, and 3. a *decoder* that reconstructs the image encodings back into the original image space. Additionally, skip connections are introduced from the encoder to the decoder to preserve contextual features during the reconstruction process.

#### 3.1.1 Image encoder design

In the image encoder, the input image is first divided into patches, which are then processed through convolutional projections. These projections are followed by the addition of sinusoidal positional embeddings to retain spatial information Vaswani et al. (1). The patched image features are subsequently passed through 12 transformer blocks that perform self-attention and convolution operations to encode the image, capturing both local and global dependencies.

Formally, the self-attention is defined in Equation 1

(1) $$\text{Attention}\;(Q,K,V)=Z+\text{softmax}\left(\frac{QK^{T}}{\sqrt{d_{k}}}\right)V$$

where Z is the input patch from the previous layer, Q, K, and V represent the query, key, and value vectors, and $d_{k}$ is the dimensionality of the key vector.

The outputs of the self-attention mechanism are concatenated to form the multi-head attention (MHA) (as shown in the Equation 2)

(2) $$\text{MHA}\;(Q,K,V)=\text{Concat}\;({\text{Head}}_{1},{\text{Head}}_{2},\ldots ,{\text{Head}}_{n})$$

where each ${\text{Head}}_{i}$ is computed as in Equation 1.

In the standard transformer block, the MHA output is typically passed through a *multi-layer perceptron* (*MLP*) followed by a residual connection:

(3) $${\mathrm{ViT}}_{\mathrm{mlp}}=\mathrm{MLP}\;(\mathrm{MHA}(Q,K,V))+\mathrm{MHA}\;(Q,K,V)$$

However, in AttnFnet, we replace the *MLP* with convolutional projections, allowing the encoder to refine features more quickly while maintaining spatial hierarchies:

(4) $${\mathrm{ViT}}_{\mathrm{conv}}=\mathrm{Conv}(\mathrm{MHA}(Q,K,V))+\mathrm{MHA}(Q,K,V)$$

Skip connections are introduced between intermediate transformer layers and the decoder block to help retain high-resolution details.

Both model variants were evaluated:

• **ViT-mlp**: AttnFnet with an MLP feed-forward network in the transformer block, as shown in Equation 3.
• **AttnFnet**: AttnFnet with convolutional projections in the transformer block, as shown in Equation 4.

#### 3.1.2 Image decoder

The decoder reconstructs the encoded image representations by upsampling them through successive deconvolution layers. These layers progressively increase the spatial resolution until the original input size is restored. To preserve critical image details, skip connections from the encoder are incorporated, allowing the decoder to combine low-level feature maps with upsampled features and enhance high-resolution reconstruction. Unlike the encoder, the decoder is designed to be lightweight, focusing on upsampling the encoded features.

### 3.2 Training objective

The training objective is inspired by the Pix2Pix framework (6), where we employ a conditional GAN (cGAN) architecture with a PatchGAN discriminator. The PatchGAN discriminator distinguishes between real and generated image pairs, ensuring that local image details are accurately predicted while maintaining global consistency in the generated pressure maps.

The total training objective aims to optimize both the discriminator and generator losses. The discriminator loss $L_{D}$ is defined in the Equation 5.

(5) $$\begin{array}{rl} L_{D} & =-[E_{x,y}[y_{\text{real}}\cdot \log(D(x|y)))]+E_{x,y}[(1-y_{\text{real}})\cdot \log(1-D(x|y))]\\ & \;+E_{x}[y_{\text{gen}}\cdot \log(D(x|G(x))))]+E_{x}[(1-y_{\text{gen}})\cdot \log(1-D(x|G(x)))]] \end{array}$$

where x is the input depth image, y is the ground truth pressure distribution map, and G(x) is the generated pressure map from the generator. The first two terms evaluate how well the discriminator identifies real image-label pairs, while the last two terms penalize the discriminator for misclassifying generated pressure distribution maps as real. Here, $y_{\text{real}}$ refers to the label for real pressure maps, and $y_{\text{gen}}$ refers to the label for generated pressure maps.

The generator loss $L_{G}$ combines the adversarial loss with perceptual loss (as shown in Equation 6), encouraging the generated images to be both realistic and similar to the ground truth:

(6) $$L_{G}=-E_{x}[\log(D(x|G(x))))]+\lambda \cdot E_{x,y}[L_{SSIML2}]$$

Here, λ is a regularization constant that balances the contributions of the adversarial and perceptual losses.

The perceptual similarity L2 loss $L_{\text{SSIML2}}$ combines the Structural Similarity Index Measure (SSIM) loss with the mean squared error (MSE) loss:

(7) $$L_{\text{SSIML2}\;}(x,y)=\alpha \cdot (1-\text{SSIM}(y,G(x)))+\beta \cdot \|y-G(x){\|}_{2}^{2}$$

where α and β are weighting factors for the SSIM and MSE components, respectively.

The SSIM between two images a and b is defined as:

(8) $$\text{SSIM}\;(a,b)=\frac{\left(2\mu_{a}\mu_{b}+C_{1})(2\sigma_{ab}+C_{2}\right)}{\left(\mu_{a}^{2}+\mu_{b}^{2}+C_{1})(\sigma_{a}^{2}+\sigma_{b}^{2}+C_{2}\right)}$$

where:

• $\mu_{a}$ and $\mu_{b}$ are the mean values of a and b, respectively.
• $\sigma_{a}^{2}$ and $\sigma_{a}^{2}$ are the variances of a and b.
• $\sigma_{ab}$ represents the covariance between a and b.
• $C_{1}$ and $C_{2}$ are constants to stabilize the division when the denominator is small.

By combining SSIM with pixel-level MSE loss, the model is encouraged to maintain structural similarity while optimizing pixel-wise accuracy, which helps to produce more perceptually faithful reconstructions.

### 3.3 Training strategy

#### 3.3.1 Dataset

The proposed model was evaluated on an open-source dataset from Liu et al. (8). The dataset includes depth images of 102 healthy subjects (28 female) in 45 unique poses, each lying on a hospital bed. The poses are classified into three primary postures: supine, left-side lateral, and right-side lateral. The data were split into training (data from n=61 subjects), validation (data from n=20 subjects), and test sets (data from n=21 subjects). The training data did not include poses with blanket covers or synthetic data.

The model used only depth information to predict pressure distributions and did not utilize any Supplementary Material from the dataset. However, the model uses Occlusion Free Depth Images (OFDI), which are noise-free, cropped depth images containing all data points from the human surface (32), and Pre-processed Pressure Distribution (PPress). The PPress involves reducing the image resolution to 27×64 and applying a Gaussian filter (σ=1.4) to diminish noise and smooth the pressure images (33). This preprocessing step is conducted to assess its impact on prediction accuracy and to facilitate comparison with (10).

#### 3.3.2 Training settings

All networks were trained using the same settings, except for the learning rate η. The Adam optimizer (34) was employed for optimization, using a learning rate of $\eta =2\times 10^{-4}$ for the U-Net model and $\eta =1\times 10^{-4}$ for AttnFnet. The initial decay rates (β) for the Adam optimizer were set to $\beta_{1}=0.5$ and $\beta_{2}=0.999$. All the optimizer parameters were the same for the discriminator and generator. All models were trained until 90 epochs with a batch size of 1.

For conditional GAN training, a regularization constant λ=100 was used in the generator loss, with weighting factors α=300 and β=1 in the perceptual similarity L2 loss (Equation 7). Since image generation tasks are generally more challenging than image classification tasks, label smoothing was applied to reduce the confidence of the discriminator, setting the label for generated pressure distribution maps to $y_{\text{gen}}=0.1$ and the label for real distribution maps to $y_{\text{real}}=0.9$.

#### 3.3.3 Evaluation metrics

• **Pixel Prediction Accuracy (PPA)**: Pixel Prediction Accuracy (PPA) is described by the ratio of the total correctly predicted pixels to the total number of pixels Equation 9.
  (9) $$\text{PPA}=\frac{\text{Number of True Predictions}}{\text{Number of Total Pixels}}$$
• **Structural Similarity Index Measure (SSIM)**: Structural Similarity Index Measure (SSIM) is defined in Equation 8.
• **Fréchet Inception Distance (FID)**: Defined by Heusel et al. (35). Fréchet Inception Distance (FID) is calculated from the features, extracted using the pre-trained inception-V3 model trained on the imagenet dataset.
• **MSE**: Calculates the average squared difference between the estimated values ${\hat{Y}}_{i}$ and the actual values $Y_{i}$ across all the data points n, Equation 10.
  (10) $$\mathrm{MSE}=\frac{1}{n}\sum_{i=1}^{n}{\left(Y_{i}-{\hat{Y}}_{i}\right)}^{2}$$
• **Peak Signal-to-Noise Ratio (PSNR)**: PSNR Measures the ratio between the maximum possible power of a signal and the power of corrupting noise, defined in (36).
• **Posture Intersection Over Union (IOU)**: The largest area of pressure higher than the threshold in actual pressure distribution is $A_{y}$ and the largest area of pressure exceeding the threshold in predicted pressure distribution is $A_{\hat{y}}$. posture Intersection Over Union (IOU) is defined by Equation 11.
  (11) $$IOU\;(A_{y},A_{\hat{y}})=\frac{A_{y}\cap A_{\hat{y}}}{A_{y}\cup A_{\hat{y}}}$$

The metrics–Mean Pixel Prediction Accuracy (MPPA), Mean Structural Similarity Index (MSSIM), Mean Fréchet Inception Distance (MFID), MSE, Mean Peak-Peak Signal-to-Noise Ratio (MPSNR), and Posture Mean Intersection Over Union (MIOU) are the average values across the test data. These metrics provide a comprehensive evaluation of the models in terms of both pixel-level accuracy and perceptual quality.

## 4 Results

We evaluated the performance of the proposed AttnFnet model and compared it with implementations of U-Net, BPBnet, and BPWnet (9, 10). The variation of AttnFnet—ViT-mlp was also assessed to determine the impact of the convolutional projections in the transformer blocks.

### 4.1 Quantitative evaluation

Table 1 compares the MPPA, MSSIM, MFID, MSE, and MPSNR scores calculated on test data from U-Net and AttnFnet model predictions. The results indicate that AttnFnet achieves higher MSSIM and MPSNR scores, as well as lower MSE scores, compared to U-Net, ViT-mlp, BPBnet, and BPWnet. Notably, AttnFnet outperforms U-Net by 15% in terms of MSE.

**Table 1:** MPPA, MSSIM, MFID, MSE, and MPSNR metrics comparison with the state-of-the-art on the test data.

Model	MPPA	MSSIM	MFID	MSE	MPSNR
U-Net	**0.6658**	0.7958	0.4615	0.000433	34.4185
AttnFnet	0.6142	**0.8291**	0.3475	**0.000368**	**35.0508**
ViT-mlp	0.5112	0.7968	**0.2393**	0.000426	34.2621
BPBnet (10)	0.0078	0.0204	160.58	0.00567	22.5927
BPWnet (10)	0.5244	0.6331	1.6335	0.00405	24.1364

Bold values denote the best score for each metric.

**Figure 2:**
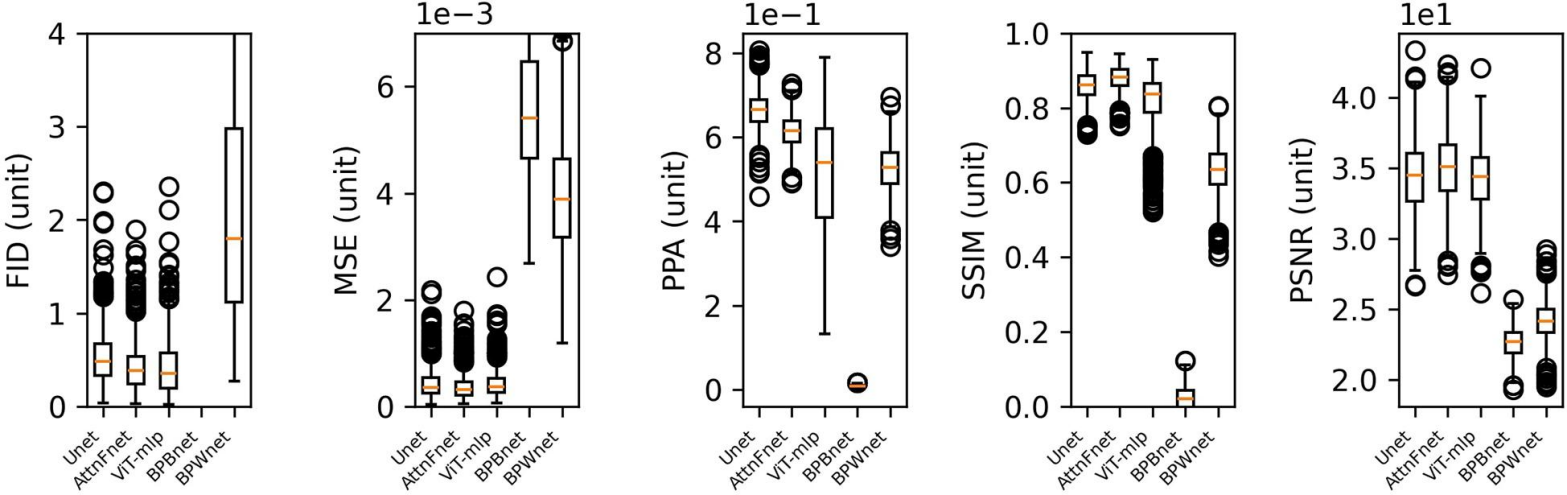
Box plot representation of FID, MSE, PPA, and SSIM metric scores obtained from test predictions.

Figure 2 presents box plots of the FID, MSE, PPA, and SSIM metrics for the U-Net, AttnFnet, ViT-MLP, BPBnet, and BPWnet. AttnFnet shows a narrower Interquartile Range (IQR) and lower median values in MSE, indicating more consistent performance. U-Net demonstrates higher median and IQR in PPA, suggesting superior pixel-level accuracy. However, AttnFnet achieves better SSIM scores, reflecting higher structural similarity with the actual pressure distributions. AttnFnet version of ViT-mlp has a lower MFID score, but AttnFnet has a narrower IQR than any other method. The proposed methodology outperforms BPBnet and BPWnet in all metrics.

### 4.2 Effects of image pre-processing

The pressure distributions were converted to kPa by multiplying calibration factors from the dataset (8) with the pressure distributions, and the MSE was recalculated. Table 2 presents the overall MSE across the test dataset for models trained on three cases: 1. raw depth images as input and raw pressure images as ground truth, 2. Occlusion Free Depth Images (OFDI) inputs, and 3. combined OFDI input with PPress ground truth.

**Table 2:** Overall MSE comparison of U-Net, AttnFnet, and ViT-mlp model predictions on test subjects, with results compared against BPWnet and BPBnet models proposed by Clever et al. (10). Models were trained on three different cases: 1. raw depth input with raw pressure ground truth, 2. OFDI input with raw pressure ground truth, and 3. combined OFDI input with PPress ground truth. MSE values are derived from rescaled pressure distributions in kPa.

Model	OFDI	PPress	MSE ↓ (${\text{kPa}}^{2}$)
U-Net			2.7871
	×		2.5694
	×	×	0.7950
AttnFnet			2.5354
	×		2.3333
	×	×	**0.6884**
ViT-mlp			2.6614
	×		2.5023
	×	×	0.8091
BPBnet (10)	×	×	0.772
BPWnet (10)	×	×	1.155

Bold values denote the best score for each metric.

Using Occlusion Free Depth Images (OFDI) and Pre-processed Pressure Distribution (PPress) resulted in a 33% greater reduction in error compared to using raw depth images. Notably, AttnFnet achieved better results in this scenario.

### 4.3 Qualitative analysis

**Figure 3:**
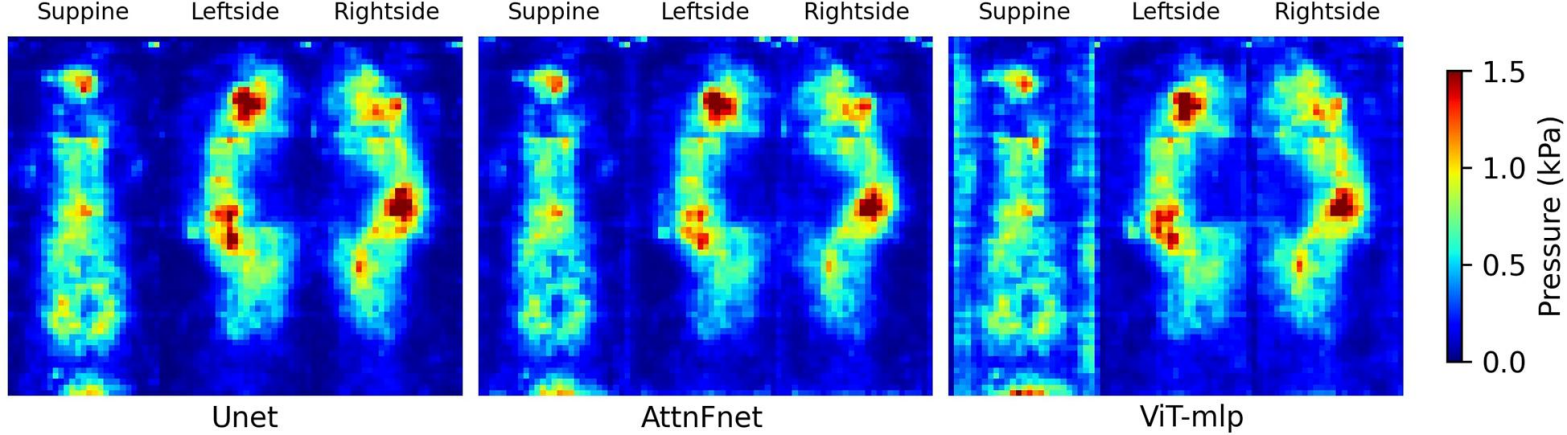
Visual representation of the pressure deviations in supine, left-side lateral, and right-side lateral postures. The heat map is constrained between pressure deviation values of 0 and 1.5 kPa.

Figure 3 shows the average deviations for three different postures—supine, lateral left-side, and lateral right-side -, comparing the U-Net, AttnFnet, and ViT-mlp models. Absolute deviations were calculated by taking the absolute pressure difference between the actual and predicted pressure distribution and averaging it over the test dataset.

**Figure 4:**
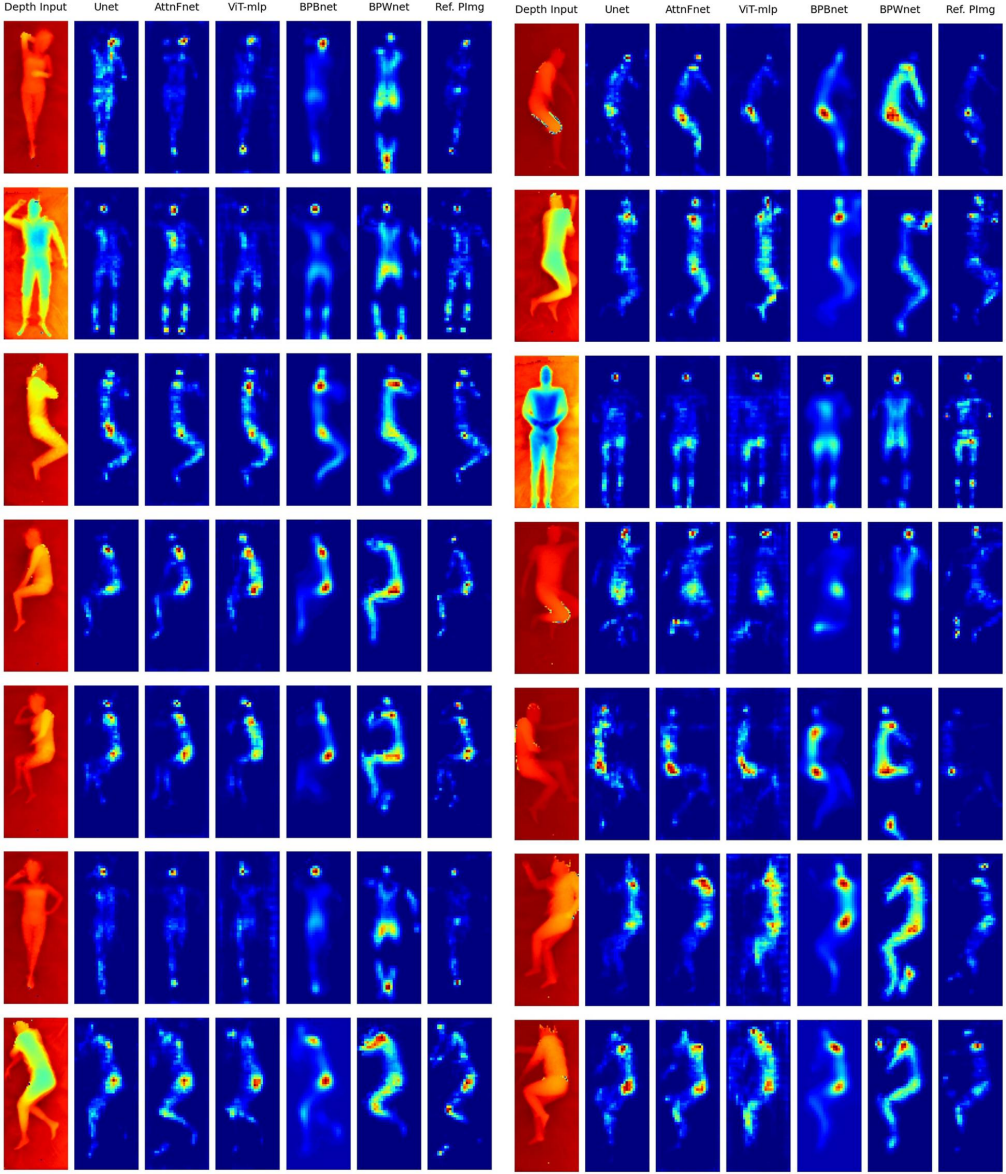
Visual representation of the predicted pressure distributions using five different models and their comparison to the reference pressure image (Ref. PImg). Occlusion Free Depth Images (OFDI)s were used as input to the models. Each row represents a different depth input to the models. In the pressure distribution images, blue indicates low-pressure regions, and red indicates high-pressure regions. In the depth images, red indicates higher depth and blue indicates lower depth values.

A visual comparison of the predicted pressure distributions using U-Net, AttnFnet, ViT-mlp, BPBnet, and BPWnet models, against the reference pressure images, shown in Figure 4. AttnFnet produced more accurate posture representations compared to U-Net, ViT-mlp, BPBnet, and BPWnet. AttnFnet’s predictions were more closely aligned with the actual pressure distribution. U-Net often struggled with pressure distribution on the leg and head side, while ViT-mlp tended to predict higher pressure values around the edges of the human body. BPBnet produces blurry results due to its pixel loss reduction, while BPBnet doesn’t produce blurry results but overestimates pressure values and couldn’t outperform AttnFnet.

Notably, all models consistently overestimated pressure values compared to the actual distribution in the facial and pelvic regions.

### 4.4 Weight estimation

**Figure 5:**
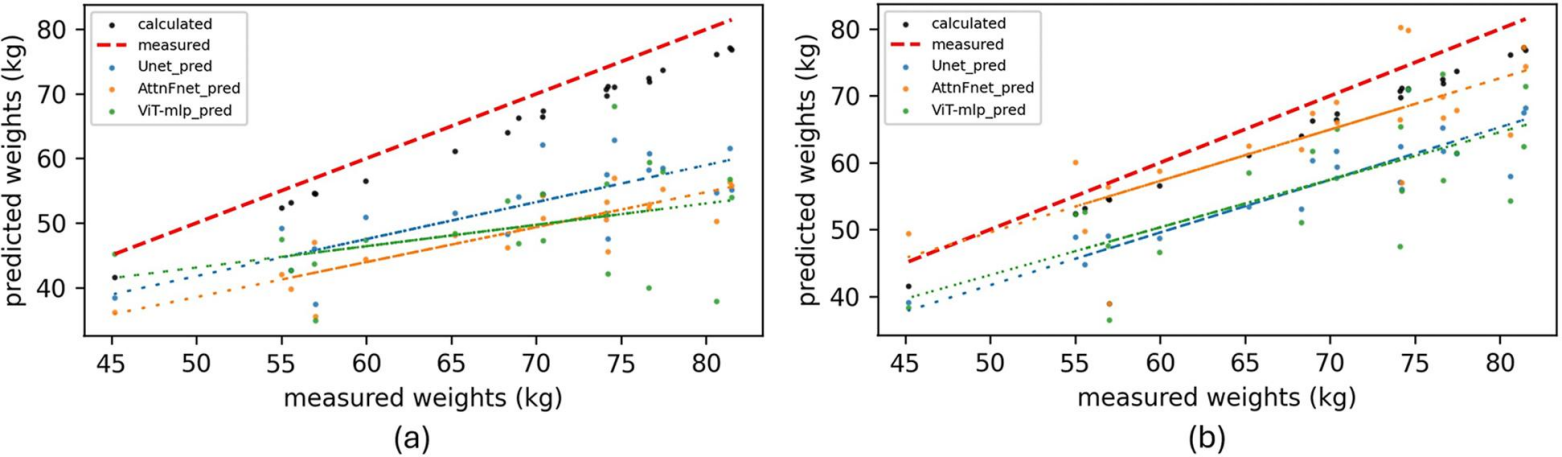
Scatter plots representing the errors in estimated weights (kg) of test subjects. Comparison between calculated weights (kg) from predicted pressure distributions, calculated weights from actual pressure distributions (kg) (Black points), and actual measured weights (kg) (red dashed line). **(a)** Estimated weights using raw depth images as input. **(b)** Estimated weights using cleaned depth images (OFDI) as input to the proposed models.

By using the predicted pressure distributions and the known area of each sensor, the normal force on the mattress was calculated (see Supplementary Material, Section 1). This force provided an approximate estimate of the test subjects’ weights. Figure 5 shows scatter plots comparing the estimated weights of each participant based on actual and predicted pressure distributions from the proposed models.

Figure 5 shows that the use of OFDIs improves the performance of AttnFnet and ViT-mlp, leading to more accurate pressure distributions and better weight estimations, as evidenced by the fitted line of AttnFnet’s estimated weights.

Table 3 shows AttnFnet performs best in Posture MIOU while BPWnet gives better weight estimation among all models.

**Table 3:** Mean absolute weight difference between the calculated weight from the predicted pressure profile and the actual measured weight. Weight is computed using both raw and OFDI inputs with the U-Net, AttnFnet, and ViT-mlp models. The last column shows the Posture Mean Intersection Over Union (MIOU) from predictions using each method.

Method	Mean absolute weight difference (kg)		Posture MIOU
	Raw input	OFDI input	
U-Net	**12.65**	12.30	0.7346
AttnFnet	16.50	6.71	**0.7515**
ViT-mlp	21.63	12.19	0.4910
BPBnet (10)	–	–	0.7329
BPWnet (10)	–	**5.64**	0.6566

Bold values denote the best score for each metric.

## 5 Discussion

The proposed AttnFnet model effectively infers body pressure distribution from a single depth image. The AttnFnet architecture leverages self-attention mechanisms to generate more refined features during image encoding in latent space, offering improved performance over U-Net. The results demonstrate that the proposed method outperforms state-of-the-art methods.

### 5.1 Effectiveness of SSIML2 loss function

The use of the combined Structural Similarity Index Measure and L2 norm loss (SSIML2 loss) provided stable training and better performance. When the model was trained using only the L2 norm loss with adversarial loss, it exhibited signs of mode collapse, and the validation MSE loss started increasing after 40 epochs when the MSE could not be reduced further (see Supplementary Material, Section 2). Training with the L2 norm loss resulted in a 130% increase in MSE and a 31.17% reduction in SSIM compared to the model trained with SSIML2 loss.

### 5.2 Robustness to noisy data

As shown in Table 2, the proposed model successfully generated pressure distributions even from noisy raw data, with significantly reduced error when using OFDI and PPress. The ability to handle raw depth images and generation of pressure distribution without introducing blurring demonstrates the robustness of the proposed method (more in Supplementary Material, Section 2). This suggests that while the model is capable of learning from noisy input, preprocessing steps can enhance its predictive accuracy.

### 5.3 Plausibility of pressure distributions

The results from Table 3 and Figures 3–5, show that AttnFnet’s attention over features helps the model produce more plausible feature distributions compared to other models. In Figure 4, AttnFnet outperforms other methods in terms of posture representation and visual accuracy of the pressure distributions. Specifically, in Figure 3 it is evident that near the hip and head areas—where all methods tend to overestimate pressure values—AttnFnet tends to reduce overestimation.

Moreover, while Table 3 and Figure 5 show that weight estimation from U-Net predictions does not improve significantly with preprocessed inputs, AttnFnet’s performance increases notably. This indicates that AttnFnet learns the relationship between depth representation and pressure distribution more effectively through its attention mechanism. However, calculated weights from all methods exhibit some scatter and do not outperform the BPWnet from Clever et al. (10). This disparity is because Clever et al. (10) utilized a separate pre-trained network “Betanet,” to estimate the mass and height of the subject and incorporate this information into the loss function to improve results. In contrast, our method does not use any Supplementary Material during training and relies solely on features from Occlusion Free Depth Images (OFDI).

Table 3 also shows the mean posture Intersection over Union (IOU), with the ViT-mlp method having the lowest score. The ViT-mlp variant tends to generate higher pressure values at the edges of the human posture, resulting in a visual representation that appears wider than the reference image. This is evident in Figures 3, 4.

As shown in Figure 4, BPBnet exhibits blurred predictions due to its training strategy based on pixel reduction losses (L1 and L2 losses). This approach tends to average pixel values, which can result in improved MSE performance but fails to yield better results across other evaluation metrics. In contrast, BPWnet does not exhibit blurring; however, it tends to overestimate pressure values compared to the actual distributions and fails to generate postures superior to those of the AttnFnet model, as evident in Figure 4.

### 5.4 Model performance and capabilities

The proposed model achieved better performance across several evaluation metrics, including MFID, MSSIM, MSE, and MPSNR, compared to previous methods. Among the variants of AttnFnet, the ViT-mlp version showed the best MFID score. This improvement is partly due to how the FID score is calculated, which heavily depends on the specific version of the ImageNet dataset and the pre-trained Inception-V3 model employed for feature extraction. FID measures how closely the generated images resemble real ones by comparing high-level features, focusing on the mean and covariance of these features in both real and generated images. However, a lower FID score does not necessarily indicate identical pressure distributions; it also accounts for the diversity of generated data (35). Therefore, it is most reliable when evaluating realistic RGB images.

The self-attention mechanism in the AttnFnet model captures meaningful relationships between feature embeddings, producing features that encompass both local and global information. Skip connections in the architecture help the model retain high-resolution features and improve performance by facilitating gradient flow and feature reuse (see Supplementary Material, Section 2). The proposed method initializes attention weights from Segment Anything Model (SAM) (3), which aids better weight initialization even though Segment Anything Model (SAM) was trained on a different objective. While we did not perform a comparative analysis of the model’s performance without transfer learning, prior work by Raghu et al. (23) supports the argument by comparing ViTs to ResNet models with and without pretrained weights.

Despite the slower learning rate, AttnFnet achieved a lower validation loss faster than U-Net (see Supplementary Material, Section 2). This suggests that the transformer/based architecture of AttnFnet is more efficient in capturing the complex relationships in the data, even with a reduced learning rate.

Overall, the experimental results validate that the AttnFnet model gives better performance in inferring pressure distributions from depth images. The incorporation of the SSIML2 loss function, robustness to noisy data, and effective use of self-attention mechanisms contribute to the model’s improved accuracy and reliability. Additional performance measures can be found in the Supplementary Material.

## 6 Future work and limitations

Although the proposed method outperforms other models still lacks clinical validation and can generate certain data dependency. To generalize the model and reduce data dependency, future work involves the collection of diverse datasets with patients and healthy controls.

Challenging errors, such as a person having a lipoma beneath the skin tissue or a very complex human posture, may cause the model to predict inaccurate pressure distributions. The authors expect future work towards incorporating physical plausibility constraints and informed learning approaches during training to reduce errors and ensure physically plausible pressure distributions.

The proposed model can be adapted for generalized image translation tasks. The authors expect future work toward the evaluation of the proposed method compared to state-of-the-art image translation methods.

Model employs cGAN to improve pressure prediction; however, GANs are sensitive towards hyperparameters and difficult to train. The authors will guide future work towards, conditional diffusion process to improve prediction even further.

## 7 Conclusion

In conclusion, we have proposed a self-attention-based deep neural network, AttnFnet, to translate depth images into pressure images. We evaluated two variations of the proposed architecture—ViT-mlp and AttnFnet—against state-of-the-art methods. The proposed method outperforms the existing methods, achieving 91% reduction in MSE and 30% increment in MSSIM score compared to the state-of-the-art BPWnet. It also outperforms existing methods in qualitative analysis of the uncovered systematic lying postures of the real test subjects, demonstrating its potential for accurate pressure distribution prediction from depth images.

These findings can help detect and prevent early pressure ulcers by identifying risk areas of a patient lying on a bed. The current publicly available dataset is limited to supine and lateral postures; so future works involve extending it towards prone and sitting postures to cover diverse risk-affected areas.

## Data Availability

The original contributions presented in the study are included in the article/Supplementary Material/Github Repository (37), further inquiries can be directed to the corresponding author/s.
