# Supplementary material for: ATTNFNET: feature aware depth-to-pressure translation with cGAN training
Source: Front Med Technol. 2025 Sep 16;7:1621922. doi: 10.3389/fmedt.2025.1621922 (PMC12479316; doi:10.3389/fmedt.2025.1621922)
Supplement: Supplementary file 1 [file Datasheet1.pdf]

Supplementary Material

1 BODY MASS CALCULATION

Ideally, the sum of all pressure values multiplied by their corresponding areas equals a person's body weight. However, when recording pressure using a pressure mat on a soft mattress, and placing a cloth between the pressure mat and the human subject, deviations in the measured pressure values occur (1).

The approximate body mass (BM) of a subject lying on a bed is calculated by the following equation:

$$\text{BM} = \sum_{t=1}^{K} \frac{P_t A_t}{g} \tag{S1}$$

where P_t is the pressure at taxel t, A_t is the area of a single taxel, and g is the gravitational acceleration $(9.8m/s^2)$. The area of a single taxel is calculated using its height and width, with $H = W = 1.016\,\text{cm}$.

Each subject performed 45 unique poses, so the average body mass (ABM) of one subject is calculated using equation S2

$$\text{ABM} = \frac{1}{45} \sum_{i=1}^{45} BM_i \tag{S2}$$

2 ADDITIONAL RESULTS

1

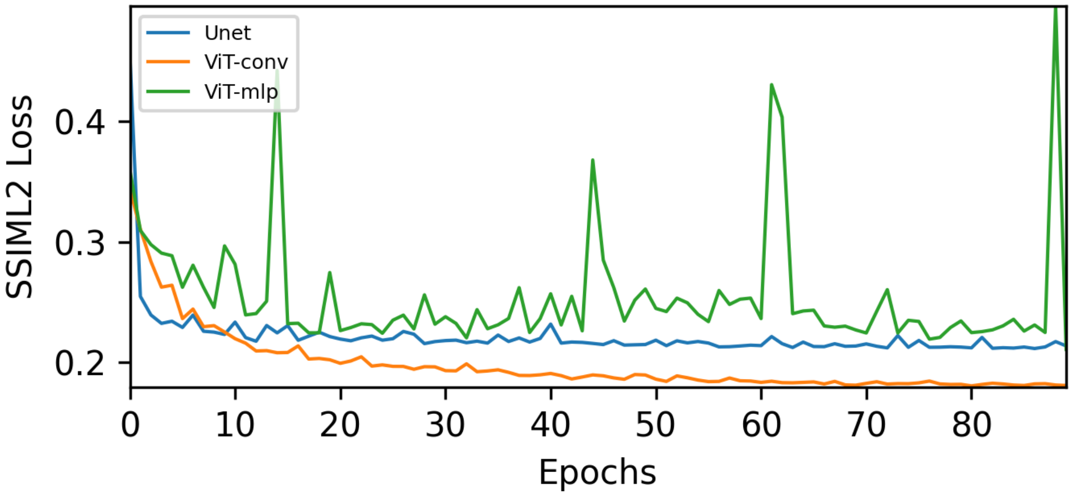

Figure S1: Validation history of the U-Net, AttnFNet, and ViT-mlp models on the validation set during the model training.

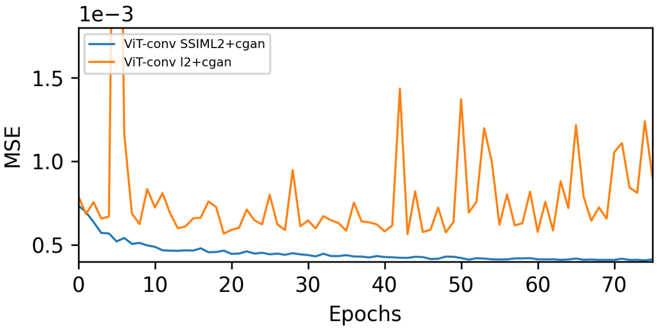

Figure 2a: Validation Mean Squared Error (MSE) loss

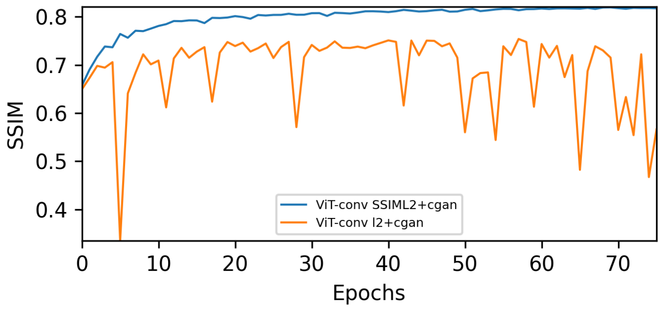

Figure 2b: Validation Structural Similarity Index Measure (SSIM) score

Figure 2: Validation Mean Squared Error (MSE) loss and Structural Similarity Index Measure (SSIM) metric comparison during model training. Attention Feature Network (ATTNFNET) l2+cgan (red line) indicates the ATTNFNET variant trained on MSE loss with adversarial loss, and ATTNFNET SSIML2+cgan (blue line) indicates the ATTNFNET variant trained using SSIML2 loss with adversarial loss.

3

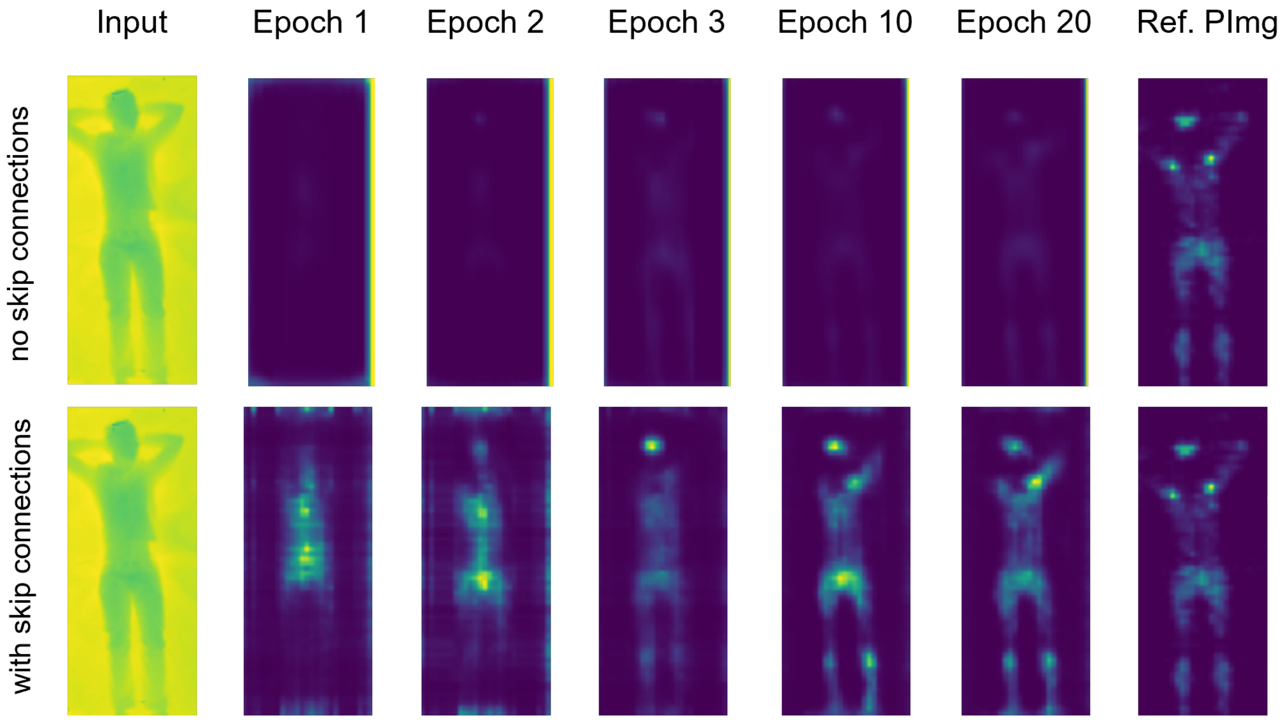

Figure S3: Training predictions on validation batch, comparing the model ATTNFNET ATTNFNET variant with skip and without skip connections. the first column represents the input depth image, and the last column represents the reference pressure image.

Figure S1 illustrates the validation SSIML2 loss for U-Net, ViT-mlp, and ATTNFNET models over 90 epochs. As discussed in the main paper, ATTNFNET achieves lower loss more quickly than U-Net and ViT-mlp.

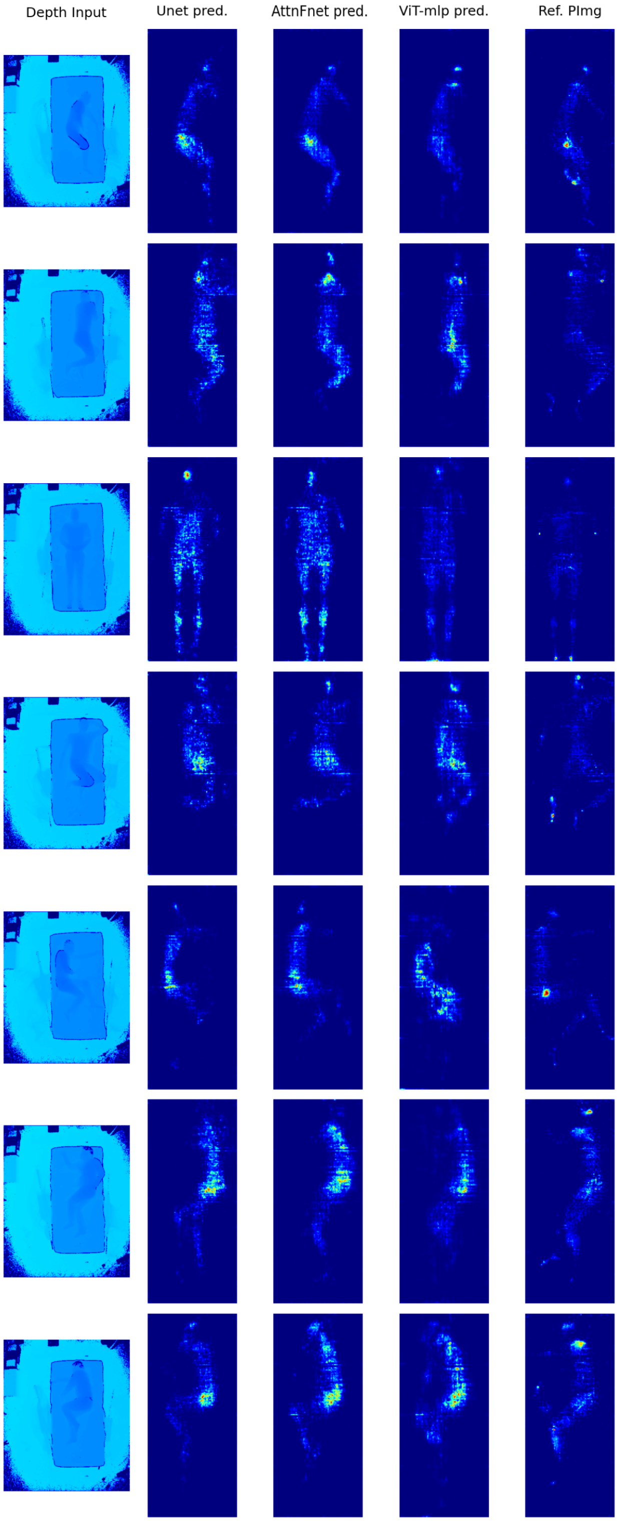

Figure 4a: raw depth image as input

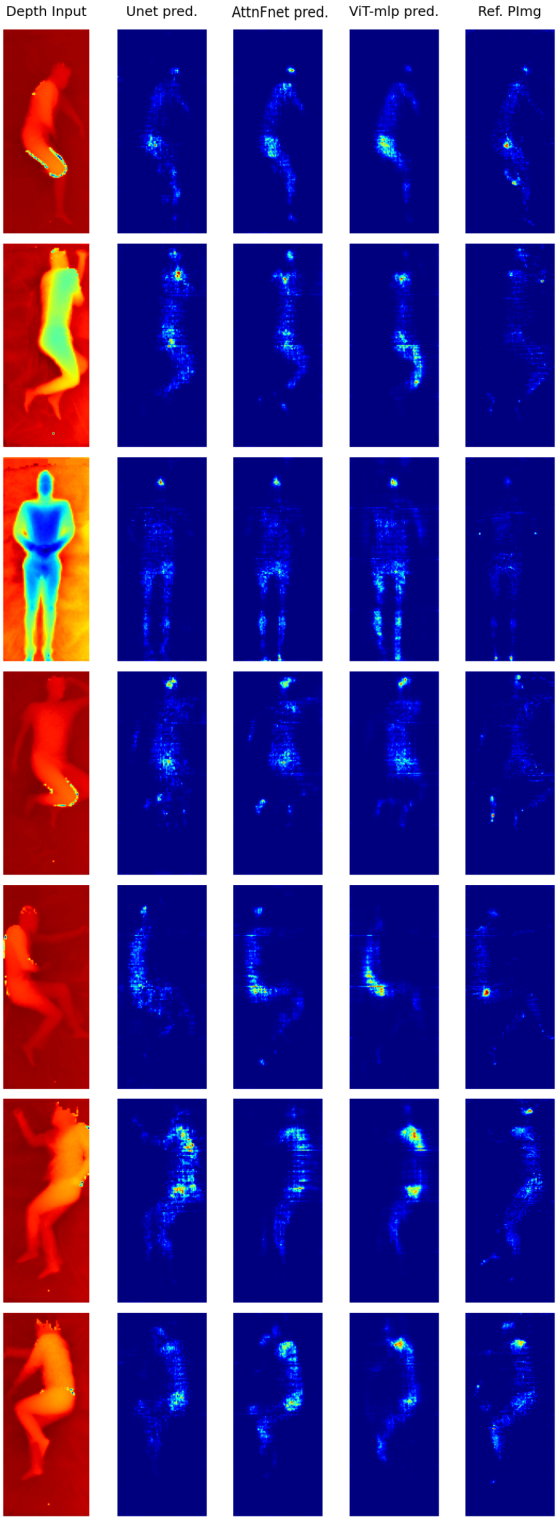

Figure 4b: OFDI as input

Figure 4: Visual representation of the predicted pressure distributions using three different models and their comparison to the reference pressure image (Ref. PImg). **(A)** raw depth image (416×512) was used as input to the models without Pre-processed Pressure distribution (PPRESS), **(B)** Occlusion Free Depth Images (OFDI) (128×54) was used as input to the model without PPRESS. Each row represents a different depth input to the models. In the pressure distribution images, blue indicates low-pressure regions, and red indicates high-pressure regions. In the depth images, red indicates higher depth and blue indicates lower depth values.

Figure 2 compares validation MSE and SSIM metrics when the model was trained using L2 norm loss and SSIML2 loss. Training with SSIML2 loss results in more stable MSE convergence and improved SSIM scores. After 40 epochs, models trained with L2 norm loss began to collapse, leading to increased validation loss.

Figure S3 demonstrates the training predictions of ATTNFNET on a validation batch across different epochs, highlighting the impact of skip connections in the architecture. Without skip connections, prediction quality drops significantly, producing pressure distributions with very low-pressure values. Skip connections enable faster distribution generation and help the model stay aligned with the discriminator.

Figure 4 compares the predicted pressure distributions from U-Net, ATTNFNET, and ViT-mlp models. Predictions using raw depth images as input (without PPRESS) are shown in figure 4b, while figure 4b also shows predictions using OFDI as input, similarly without Figure PPRESS.

3 MODEL CONFIGURATIONS

ATTNFNET utilizes 768 embedding dimensions for patch projections. Input images are resized to 512×512 and divided into patches of size 16×16. The model employs 12 attention heads for calculating attention scores and outputs. Feed-forward networks use the 'Gelu' activation function. The model uses the 'softmax' activation function at the last layer to bind the output range between 0 and 1.

4 MODEL PARAMETERS AND TRAINING COST

The training configurations and resource requirements for U-Net, ATTNFNET, BPBnet, and BPWnet are summarized in Table S1. All models were trained for 93 epochs on 3,000 images using a batch size of 1, employing Distributed Data Parallel (DDP) on a single node with two Tesla V100-PCIE-32GB GPUs. Carbon emissions were calculated assuming a carbon efficiency of 0.29 $kgCO_2$/kWh.

Table S1. Overview of the resources used during U-Net and ATTNFNET training

Metric	U-Net	ATTNFNET
Number of Parameters	117,324,416	143,635,940
Model Parameters Size (MB)	447.56	547.93
Training Duration (hrs)	4.86	11.42
Training Speed (sec/epoch)	170	400
Memory Usage (GB/GPU)	3.5	9.5
Power Usage per GPU (W)	100	150
GPU Temperature (°C)	45	75
Total Energy Consumption (kWh)	0.97	3.45
Carbon Emissions ($kgCO_2$)	0.28	1.00

The BPBnet and BPWnet implementations exhibited higher memory consumption. BPBnet required approximately 23 GB of GPU memory, while BPWnet utilized around 17 GB, both measured with a batch size of 128. Training took almost 12 hours on NVIDIA RTX 3090 GPU (2).

5 DISCRIMINATOR ARCHITECTURE

Figure S5 depicts the PatchGAN-like discriminator architecture used to distinguish real from fake images during training. A patch size of 62×62 is employed to compare image distributions.

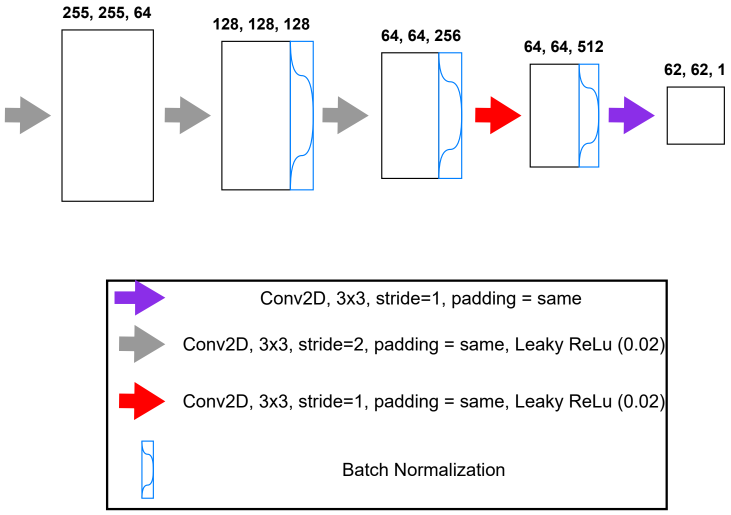

Figure S5: PatchGAN-like discriminator architecture for input size of $512 \times 512 \times 2$.

REFERENCES

1 .Clever HM, Erickson Z, Kapusta A, Turk G, Liu CK, Kemp CC. Bodies at rest: 3d human pose and shape estimation from a pressure image using synthetic data. *2020 IEEE/CVF Conference on Computer Vision and Pattern Recognition (CVPR)* (2020), 6214–6223. doi:10.1109/CVPR42600.2020.00625.

2 .Clever HM, Grady PL, Turk G, Kemp CC. Bodypressure - inferring body pose and contact pressure from a depth image. *IEEE Transactions on Pattern Analysis &; Machine Intelligence* **45** (2023) 137–153. doi:10.1109/TPAMI.2022.3158902.
